# A Fortuitous Syncope. The pitfalls of Integrated Bipolar Defibrillator Leads

**Published:** 2008-11-01

**Authors:** Tushar V Salukhe, Ian Wright, Matthew Wright, Prapa Kanagaratnam, Mark D O'Neill

**Affiliations:** Department of Cardiac Electrophysiology, St Mary’s Hospital and Imperial College, London, UK

**Keywords:** Myopotential oversensing, implantable defibrillators, inhibition of pacing, inappropriate therapy

## Abstract

Myopotential oversensing in implantable defibrillators causing inhibition of pacing and inappropriate therapies is well described. Current literature is dominated by reports of diaphragmatic muscle as the source of such far-field oversensing. Those reporting pectoral muscle sources were invariably due to unipolar sensing circuits, incorrect DF-1 connections or inappropriate programming. We report an interesting case of pectoral muscle myopotential oversensing causing inhibition of bradycardia pacing leading to presyncope and syncope.

## Introduction

Oversensing of skeletal muscle myopotentials is well described [1-3] and is common in unipolar sensing circuits where a wide sensing field is caused by distance between electrodes (the ventricular lead tip and the generator casing [~ 100 - 150 mm]). Dedicated bipolar defibrillator leads (quadripolar leads) can overcome this problem by approximating the electrodes (up to ~ 8 mm) within the lead tip, but such constructions complicate lead design and may compromise durability [4]. Integrated bipolar leads simplify lead construction by using a portion of the distal defibrillator coil and the lead tip as pace/sense electrodes (total inter-electrode distance up to ~ 60 mm). Even this degree of electrode spacing renders integrated leads susceptible to T wave, P wave and diaphragmatic myopotential oversensing [5]. We present a case highlighting the pitfalls in integrated leads in which there was no T wave, P wave or diaphragmatic oversensing.

## Case report

A 60 year old female with hypertrophic cardiomyopathy had originally presented with syncope and documented ventricular tachycardia. A dual chamber cardioverter-defibrillator (Guidant VENTAK 1861) was implanted in June 2003 and an integrated bipolar right ventricular defibrillator lead was used (Guidant SN 0158). Electrogram amplitudes, pacing and defibrillation thresholds at the time of implantation were normal.

After the procedure, the patient was without symptoms for two years. Device interrogation during routine follow-up demonstrated low levels of noise on ventricular electrograms. There were no appropriate or inappropriate therapies although three therapies were diverted.

In Feb 2006 the patient developed persistent atrial fibrillation. Pharmacological attempts to maintain sinus rhythm were unsuccessful, she declined a left atrial ablation procedure and in May 2007 the patient underwent atrio-ventricular (AV) node ablation for ventricular rate control and was rendered pacemaker dependant. One month after AV node ablation the patient attended the cardiac device clinic complaining of recurrent episodes of dizziness and syncope during certain physical activities such as showering or washing dishes. At this time pacing thresholds, electrogram amplitudes and impedances were normal. A chest X-ray showed no radiographic evidence of loss of lead integrity.

**Figure 1:**
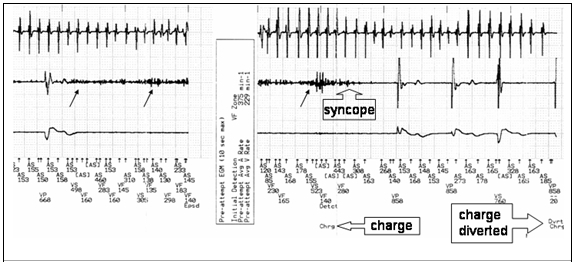
Recorded episode of diverted therapy. Atrial electrogram (top) ventricular electrogram (middle) and paced electrogram (bottom). Black arrows indicate non-physiological potentials

Device interrogation revealed 16 diverted therapies in the VF zone. One such episode is shown in Figure 1. The atrial electrogram confirms atrial fibrillation. The ventricular electrogram demonstrates a paced beat followed by potentials which are clearly not physiological. These high frequency potentials, detected within the VF zone had two consequences - inhibition of ventricular pacing and device capacitor charging. The prolonged asystole in this pacing-dependant patient eventually, and rather fortuitously, results in syncope which coincides with the disappearance of the non-physiological potentials. As a result, ventricular pacing resumes and defibrillation is diverted.

At interrogation, these potentials were not reproducible with the usual provocative manoeuvres such as rapid, deep respiration and generator agitation, but were reproducible with isometric upper limb exercise (palm apposition) and resulted in pacing inhibition and dizziness.

Our immediate management was to reduce the sensitivity to the minimum programmable setting. This was deemed safe as a short term measure as VF detection at minimal sensitivity was confirmed at defibrillation threshold testing at implantation. We advised the patient to avoid driving and provocative manoeuvres. The patient was admitted electively for re-operation. At procedure the existing lead connections to the generator were found to be secure and appropriate. Invasive lead tests were satisfactory. We elected to leave the integrated lead in situ and implant a new dedicated bipolar pacing lead (Medtronic 5076) for ventricular sensing and pacing. The new pace/sense lead was connected to the ventricular IS-1 socket on the existing device and the old integrated defibrillator lead remained connected via the DF-1 sockets. The defibrillator lead IS-1 terminal was capped and secured within the prepectoral pocket.  Subsequently, the patient's symptoms resolved and no further myopotentials were recordable.

## Discussion

The case highlights a problem specific to integrated bipolar lead technology, that is, far-field myopotential oversensing due to distance between the lead tip and distal defibrillator coil.

**Figure 2:**
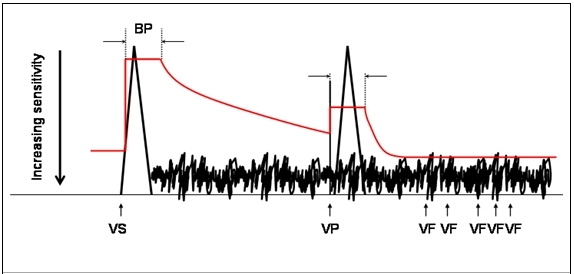
Automatic gain control (red line). See text for explanation. Blanking period (BP), ventricular sensed event (VS), ventricular paced event (VP), ventricular fibrillation detection (VF)

**Figure 3:**
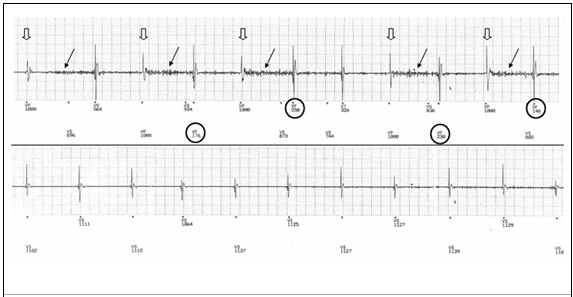
Ventricular electrograms from an implanted defibrillator interrogation. Noise (black arrows) is only detected after paced events (open arrows) resulting in events detected in the VF zone (circled, upper trace). Once base rate pacing is reduced, (lower trace), there are no paced events and as a result noise is not detected

This patient only became symptomatic when rendered pacing dependant after AV node ablation when upper body muscular movement caused pacing inhibition through myopotential oversensing. Fortunately, this consistently caused dizziness or syncope which stopped physical activity thus terminating myopotential oversensing. As a result, therapies were always diverted and pacing resumed. It is therefore curious that no inappropriate therapies were delivered prior to AV node ablation. This may simply be explained by a dynamic ventricular sensing threshold facility available in most defibrillators, in this case, automatic gain control (see Figure 2). Following a sensed ventricular event, there is a blanking period before the initial sensitivity is set to 75% of the sensed R wave amplitude. Thereafter, the sensitivity gradually increases to a set maximum (typically ~ 0.18 mV). After a paced ventricular event, the initial sensitivity is set higher (nominally ~ 3.5 mV). The subsequent rate of sensitivity increase is faster and reaches a maximum earlier. Thus, the mechanism of more frequent myopotential oversensing after becoming pacing dependant becomes clear. This phenomenon is more easily appreciated in a different patient with an implanted defibrillator in Figure 3.

Atrial and T-wave oversensing, which have also been reported with integrated bipolar leads are usually easily overcome with re-programming. True myopotential oversensing however, invariably requires re-operation. Our solution in this case was to introduce a separate dedicated bipolar pace/sense lead and retain the integrated lead for defibrillation. An alternative solution would have been to replace the lead with a dedicated bipolar defibrillator lead and either extract the integrated lead or leave it redundant [6] .

The alternative to re-operation is to reduce ventricular lead sensitivity to exclude unwanted potentials. This particular device had three sensitivity settings - "nominal", "less" and "least". Although implant testing is done at "least" sensitivity, VF re-induction at "least" sensitivity to confirm VF sensing would have been a reasonable approach (though not without risk) as changes in sensing may have occurred with lead maturation. In this case, we employed this strategy only as an interim, particularly as "least" sensitivity would not have guaranteed a resolution to myopotential oversensing, because it could not be confidently reproduced in the setting of the device clinic as myopotential interference may change with patient position and activity.

With dedicated bipolar (quadripolar) defibrillator leads available, the use of integrated leads may be questioned. Quadripolar leads are, however, not without problems. Increased pacing thresholds and sensing latency causing ventricular pseudofusion and unnecessary ventricular pacing are not uncommon [7]. More problematic is insulation failure resulting in inappropriate therapies [4].

Progress in lead technology has not paralleled the rapid advances in generator design and technology. Newer quadripolar constructions with active fixation are gaining merit but longevity data is still anticipated. Hence, to date lead choice remains at the discretion of the implanter rather than guided by evidence.
